# Transcanalicular Multidiode Laser Versus External Dacryocystorhinostomy in the Treatment of Acquired Nasolacrimal Duct Obstruction

**DOI:** 10.14744/bej.2021.04934

**Published:** 2021-12-17

**Authors:** Asker Bulut, Mehmet Gokhan Aslan, Veysi Oner

**Affiliations:** 1 Department of Ophthalmology, Istanbul Medipol University Faculty of Medicine, Istanbul, Turkey; 2 Department of Ophthalmology, Recep Tayyip Erdogan University Faculty of Medicine, Rize, Turkey; 3 Department of Ophthalmology, Dunyagoz Altunizade Hospital, İstanbul, Turkey

**Keywords:** Acquired nasolacrimal duct obstruction, external dacryocystorhinostomy, transcanalicular multdiode laser dacryocystorhinostomy

## Abstract

**Objectives:**

This study was a comparison of the outcomes of transcanalicular multidiode laser dacryocystorhinostomy (TCLDCR) and external dacryocystorhinostomy (EXDCR) treatment for patients with acquired nasolacrimal duct obstruction.

**Methods:**

Thirty-one consecutive patients who underwent TCLDCR (TCLDCR group) and 68 consecutive patients who underwent EXDCR (EXDCR group) due to acquired nasolacrimal duct obstruction were enrolled in the study. Follow-up visits were performed on the first day, and at the first week, first month, third month, sixth month, and every six months thereafter. Surgical success was defined as achievement of a patent osteotomy and a successful bicanalicular silicone intubation during the procedure. Anatomical success was defined by observation of a patent osteotomy on lacrimal irrigation, regardless of epiphora. The surgery time and intra- and postoperative complications were noted for each patient.

**Results:**

The TCLDCR group had a significantly shorter mean surgery time (27.9±5.5 minutes) compared with the EXDCR group (58.5±12.0 minutes) (p<0.001). However, the mean anatomical and functional rates of TCLDCR (58.0% and 54.8%, respectively) were significantly lower than those of the EXDCR group (94.1% and 91.1%, respectively) (both p<0.001). Two patients had “cheese wiring” damage of the lower canaliculus and 1 patient in the TCLDCR group had a full-thickness skin defect in the medial canthal region. No serious intra- or postoperative complication occurred in the EXDCR group.

**Conclusion:**

Although a TCLDCR procedure decreased the surgical time, it had a significantly lower success rate in the treatment of acquired nasolacrimal duct obstruction compared to EXDCR. The decision of the type of surgery should be made based on the cosmetic and success expectations of the patients and the presence of systemic problems.

## Introduction

Since described by Toti in 1904 and improved by Dupuy-Dutemps and Bourguet in 1921, external dacryocystorhinostomy (EXDCR) has been the standard surgical treatment of nasolacrimal duct obstruction (1). However, despite high success rates, to avoid skin scarring and reduce the operation time, different surgical techniques with endonasal and transcanalicular approaches with or without laser application have also been developed (2-4).

Endonasal lacer dacryocystorhinostomy using argon laser was introduced in 1990 (5) and the transcanalicular laser dacryocystorhinostomy was first performed in cadavers in 1992 (6). Eloy et al. (7) were the first to use diode laser for transcanalicular laser dacryocystorhinostomy in 2000. Recently, transcanalicular multidiode laser dacryocystorhinostomy (TCLDCR) is used in the treatment of patients with nasolacrimal duct obstruction, particularly for those who do not prefer EXDCR for cosmetic reasons or are not suitable to receive systemic anesthesia (8-11). In this minimally invasive approach, a laser energy with a wavelength of 980 nm is applied through a transcanalicular laser probe to open the bone window. The advantages of TCLDCR are absence of skin scarring, minimal damage to medial canthal ligament, reduced risk of hemorrhage, and decreased operation time (12).

Although TCLDCR has been demonstrated to be a safe and effective method in the treatment of nasolacrimal duct obstruction, there are few studies comparing the outcomes of TCLDCR and EXDCR (12-15). Therefore, in the present study, we aimed to compare the results of TCLDCR and EXDCR in the treatment of patients with acquired nasolacrimal duct obstruction.

## Methods

The data of 31 consecutive patients who underwent TCLDCR (TCLDCR group) and 68 consecutive patients who underwent EXDCR (EXDCR group) due to acquired nasolacrimal duct obstruction were analyzed in the study. Pre-operative detailed ophthalmic examination and irrigation of nasolacrimal duct were performed in all patients. All patients underwent a rhinoscopy before surgery to detect nasal structural anomalies. Patients who did not accept the potential skin scar and those who have systemic problems that hinder the application of general anesthesia admitted to TCLDCR group. Other patients were included in the EXDCR group. Exclusion criteria were canalicular obstruction, previous surgery or traumatic injury of nasolacrimal system, lacrimal system tumor, nasal septal deviation, and middle turbinate hypertrophy. The study was in accordance with the tenets of the Declaration of Helsinki and it was approved by the Institutional Ethics Committee (Date: December 12, 2014, Decision No: 2014/156).

EXDCR operations were performed under general anesthesia whereas TCLDCR procedures were executed under regional anesthesia with sedation. Regional anesthesia was performed through blocking infratrochlear and intraorbital nerves using 2% lidocaine with adrenaline 0.0125 mg/ml. Two percent lidocaine with adrenaline 0.0125 mg/ml impregnated sponges were placed in the middle meatus of each patient for 10 min before surgery. Topical corticosteroid (dexamethasone 4 × 1) and antibiotics (moxifloxacin 4 × 1), nasal decongestant sprays (oxymetazoline 3 × 1), and oral antibiotics (amoxicilline+clavulanic acid 2 × 1000 mg) were given to each patient for 10 days, postoperatively.

For EXDCR, a 10–12 mm straight skin incision was made medially to the angular vein. Orbicularis muscle fibers were dissected bluntly to expose the periosteum and medial canthal tendon. The medial canthal tendon was cut and separated from the bone and the lacrimal sac was exposed. After dissecting the sac from the bone, an osteotomy of approximately 15 × 15 mm was performed by Kerrison punch. Lacrimal sac and nasal mucosa flaps were formed by an H-shaped incision. Then, posterior flaps were excised, bicanalicular silicone tube (DCR set straight 23 G, Teknomek, Istanbul, Turkey) was implanted, and anterior flaps were sutured with 6/0 polyglactin absorbable suture. Finally, the orbicularis muscle and skin were also repaired with the same suture.

TCLDCR was performed using a 980 nm diode laser (Quanta system, spa model D-plus, Solbiate Olona, Italy), through a 600 μm silica polyamide laser fiber optic. The laser probe was established in the lacrimal sac through the canaliculus. The transillumination of the aiming beam was seen by a nasal endoscope in the nasal cavity across the middle turbinate. The settings were adjusted for each patient being at 10–13 Watts energy, 450 ms pulse, 450 ms pause, and contact mode. Laser energy was applied until an osteotomy with a diameter of at least 5 mm occurred. After the irrigation and removal of the debris, bicanalicular silicone tube was implanted.

Follow-up visits were performed on the 1st day, 1st week, 1st month, 3^rd^ month, 6^th^ month, and every 6 months thereafter. The follow-up time was 3 years. Surgical success was defined whenever a patent osteotomy and a successful bicanalicular silicone intubation were achieved during the procedure. Anatomical success was defined by a patent osteotomy on lacrimal irrigation regardless of epiphora. Functional success was defined by both the presence of a patent ostium on lacrimal irrigation and the complete resolution of epiphora without any episode of dacryocystitis (16). Surgery time and intra- and post-operative complications were noted for each patient.

### Statistical Analysis

SPSS version 16.0 was used for statistical analysis. Chi-square test was to compare categorical variables. Student’s t-test was used to compare continuous variables. Pearson’s correlation coefficients were used to assess relations between the variables. P<0.05 was accepted as statistically significant.

## Results

The mean age was 48.6±15.3 (range: 13–80) years in the TCLDCR group and 54.8±15.9 (range: 18–86) years in the EXDCR group. There were 23 female (74.2%) and 8 male (25.8%) in the TCLDCR group and 51 female (75.0%) and 17 male (25.0%) in the EXDCR group. There were no significant differences between the groups concerning age and gender (P1=0.0071 and P2=0.932, respectively).

The clinical characteristics of the groups are given in Table 1. The TCLDCR group had significantly shorter mean surgery time compared with EXDCR group (p<0.001). There was no correlation between age and anatomical and functional success (both p<0.05). On the other hand, the mean anatomical and functional rates of TCLDCR (58.0% and 54.8%, respectively) were significantly lower than those of EXDCR (94.1% and 91.1%, respectively) (both p<0.001). The causes of anatomical failure were cicatricial closure of the ostium (n=16/88.8%), nasal synechiae (n=1/0.05%), and DCR ostium granulomas (n=1/0.05%). The groups were similar regarding the mean silicone tube implantation time (p=0.275). Surgical success was achieved in all patients.

**Table 1.** The clinical characteristics of the patients

	TCDCR group (n=31)	EXDCR group (n=68)	p
Mean surgery time (minutes)	27.9±5.5	58.5±12.0	<0.001
Mean silicone tube implantation time (months)	3.4±0.8	3.6±0.7	0.275
Mean follow-up time (months)	12.5±4.7	31.7±10.7	<0.001
Anatomical success rate, n (%)	18 (58.0)	64 (94.1)	<0.001
Functional succes rate, n (%)	17 (54.8)	62 (91.1)	<0.001
Surgical success rate, n (%)	31 (100)	68 (100)	>0.05	

EXDCR: External dacryocystorhinostomy; TCDCR: Transcanalicular multdiode laser dacryocystorhinostomy.

Two patients had “cheese wiring” damage of lower canaliculus and one patient had full-thickness skin defect in the medial canthal region, within the 1st week postoperatively, in the TCLDCR group. Reconstruction surgery was performed for patient with skin defect and early silicon tube removal was carried out for the two patients with “cheese wiring.” No serious intra- or post-operative complication including excessive bleeding, infection, or canalicular damage occurred in the EXDCR group.

## Discussion

The current study revealed that although TCLDCR had significantly shorter surgery time, success rate was significantly lower compared to EXDCR. The TCLDCR gained popularity in the past two decades to treat nasolacrimal duct obstruction with its advantages such as absence of skin incision and scar, shorter surgery time, minimal risk of bleeding, and easy applicability under local anesthesia. However, several disadvantages of this technique including synechia and granulation tissue formation, and common canalicular damage decreases its applicability to a particular group of patients (16).

The TCLDCR was reported to have a wide range of treatment success varying between 34% and 95% (8-19). Nuhoglu et al. (8) found a success rate of 95.2% for the treatment of 42 patients with nasolacrimal duct obstruction by TCLDCR which is the highest success rate in the literature. They proposed surgeon experience and meticulous patient selection as the major factors to achieve superior results. On the other hand, Joshi et al. (18) obtained 34% success of TCLDCR without silicone tube stenting. Kaynak et al. (11) reported that 85.4% of patients treated with TCLDCR experienced complete resolution of their symptoms at the 3^rd^ month after the surgery. However, functional success rate reduced to 67.7%, 63.3%, and 60.3% at the 1st month, 1st year, and 2nd year, respectively. Pinto et al. (16) displayed that anatomical and functional success rates were 80.0% and 70.8% at 6 months; 69.3% and 61.4% at 1 year; 64.2% and 58.0% at 2 years; 56.4% and 46.2% at 3 years, respectively. In the present study, the success rate of TCLDCR was 51.6% which is apparently less than the gold standard external surgery. Nowak et al. (19) showed that the anatomical and functional success rates were 56.12% and 33.81%, respectively, at the end of 3-year follow-up. The functional success was not affected by gender, age, laser energy, or duration of the surgery. Even though all operations were performed by the same experienced surgeon (VO) with silicone tube insertion, our results indicate that patient selection seems as the main factor for surgical success. Studies investigating the characteristics of the patients with treatment failure would be beneficial to better understand underlying predispositions and to suggest a template when choosing TCLDCR for the treatment of nasolacrimal duct obstruction.

Recently, few studies compared the outcomes of TCLDCR and EXDCR procedures for the treatment of nasolacrimal duct obstructions (12-15). Yener et al. (15) revealed that the functional success rate was 93.2% in EXDCR with a follow-up of 8 years and 85.7% TCLDCR with a follow-up of 7 years. The difference in the success rate was not statistically significant. Taşkıran Çömez et al. (12) reported insignificantly lower success rate with TCLDCR in resolution of symptoms (79.4%) compared with EXDCR (89.1%). The surgery time was 22.2±4.8 min for TCLDCR and 56.3±15.7 min for EXDCR (p<0.0001). Similarly, Derya et al. (13) also found lower functional success rate with TCLDCR (68% vs. 86%) and Balıkoglu-Yilmaz et al. (14) obtained lower anatomical (76.7%) and functional (73.3%) success rates with the same surgical approach. However, both studies reported significantly shorter surgery times with TCLDCR and all patients had received local anesthesia. We also obtained significantly shorter surgery time with TCLDR under regional anesthesia. Besides, the surgical incision was inevitably prominent with EXDCR. Nevertheless, the success rate of TCLDCR was significantly lower than EXDCR. The fibroblastic effect of laser application was accused by extreme fibrosis and consequently obstruction of the new rhinostomy which may explain the lower success rate (20, 21). Moreover, the narrower bone aperture in TCLDCR compared to EXDCR might be related with lower success rate (22).

Our study found no correlation between anatomical or functional success rates and age. The results of the studies which have investigated the relation between success rate TCLDCR and age are conflicting. Akay et al. (23) found that the success rate was higher (76%) in older age group (mean age: 60.3±7.3 years) than in younger group (46%) (mean age: 21.3±3.3 years). However, Kaynak et al. (11), Nowak et al. (19), and Plaza et al. (24) found no correlation between success rate and age in consistent with our study.

As a limitation of this study, that was not possible to determine the underlying factors to explain lower surgery success with TCLDCR. Therefore, further studies evaluating the impact of different laser energy parameters and measurement of the bone aperture width may define the optimum TCLDCR technic for individual requirements.

In the current study, two patients had “cheese wiring” defect of canalicula and one patient had full-thickness skin defect, after the TCLDCR procedure. Similarly, McClintic et al. (22) reported three cases with skin necrosis and Kaynak et al. (11) described four cases with slitting punctum after TCLDCR surgery. It is particularly important to protect surrounding tissues while laser application and redundant high-energy shoots without precise focusing should be avoided to diminish such complications.

## Conclusion

The present study revealed that although TCLDCR procedure decreased the surgical time with a more esthetic outcome, it had significantly lower success rate in the treatment of acquired nasolacrimal duct obstruction compared to EXDCR. Hence, patients should be warned for possible surgical failures and the need for secondary surgeries should be carefully explained. The decision of surgical choice should be clearly discussed to balance cosmetic, functional, and surgical comfort expectations of the patients. Despite the lower surgical success, TCLDCR is still a valuable choice of treatment, particularly for those who have systemic problems that hinder the application of general anesthesia.

### Disclosures

**Ethics Committee Approval:** Recep Tayyip Erdogan University Faculty of Medicine Ethics Committee, protocol number: 2014/156, Date: 12/12/2014.

**Peer-review:** Externally peer-reviewed.

**Conflict of Interest:** None declared.

**Authorship Contributions:** Involved in design and conduct of the study (AB, MGA, VO); preparation and review of the study (AB, MGA, VO); data collection (AB, MGA, VO); and statistical analysis (AB).
